# Comparison between Specific Lumber Mobilization and Core-Stability Exercises with Core-Stability Exercises Alone in Mechanical low back pain 

**DOI:** 10.12669/pjms.301.4424

**Published:** 2014

**Authors:** Rafiq Ahmed, Syed Shakil-ur-Rehman, Fozia Sibtain

**Affiliations:** 1 Dr. Rafiq Ahmed, BSPT, PG student of PP-DPT, Riphah College of Rehabilitation Sciences, Riphah International University, Islamabad, Pakistan.; 2 Dr. Syed Shakil-ur-Rehman, BSPT, MS-MSKPT, PP-DPT, PhD Scholar, Assistant Professor, Riphah College of Rehabilitation Sciences, Riphah International University, Islamabad, Pakistan.; 3 Dr. Fozia Sibtain, BSPT, MS-MSKPT, PP-DPT, Assistant Professor, Riphah College of Rehabilitation Sciences, Riphah International University, Islamabad, Pakistan.

**Keywords:** Specific lumber Mobilization, Core Satiability Exercises, Mechanical Low Back Pain

## Abstract

**Objective**: To Compare the Specific Lumber Mobilization (SLM) techniques and Core-Stability (CS) Exercises with Core-Stability Exercises Alone in Mechanical low back pain (MLP)

**Methods**: A 6 month pretest-posttest design, quasi experimental study was conducted at department of physiotherapy Khyber Teaching Hospital (KTH) Peshawar, Pakistan. We conveniently selected a sample 40 patients and placed into two groups. The SLM techniques with CS exercises was applied in group A and CS exercises alone in group B for 6 weeks. The Oswestry Disability Index (ODI) and Visual analog scale (VAS) for mechanical low back pain were assessment tools assessed for all patients before and after 6 weeks of physical therapy intervention. Data was analyzed by SPSS and statistical test were applied at 95% level of significance determine the efficacy of both the treatments regimes and compared with each other.

**Results: **After comparison between two groups, the group A treated with specific lumber mobilization techniques shows better results in improving pain (p=0.008) and reducing physical disability (p=0.004) as compared to the group B treated with specific lumber mobilization techniques alone (pain intensity: p= 0.172 and physical disability: p=0.201).

**Conclusion: **It is concluded that patients with mechanical low back pain will show more improvement in pain and function while treated by specific lumber mobilization and core stability exercises as compared to those patients who will be treated by specific joint mobilization techniques.

## INTRODUCTION

Low back pain is a common neuro-musculo-skeletal problem affecting 40% of population worldwide at some point in their life and causes significant disability with loss of productive working hours. ^1^ The common sign and symptoms are local or radicular pain, tenderness, spam, which is aggravated by movement with loss of function. ^2^ The physical or mechanical causes of low back pain are osteoarthritis, rheumatoid arthritis, degeneration of the discs between the vertebrae or a spinal disc herniation, a vertebral fracture (such as from osteoporosis), or rarely, an infection or tumor.^3^

Diagnosis of mechanical low back pain is commonly made by physical examination, physical tests, palpation, imaging such as x-rays, MRI, and CT scan. ^4^ Medication, physical therapy, and surgery are most commonly used managements of mechanical low back pain. ^5^ The physical therapy management varies according to the patient condition, and includes; modalities, exercise therapy, manual therapy and patient’s education with a comprehensive home plan of care. ^6^

The individual effectiveness of manual physical therapy and core stability exercises therapy are evident in the management of mechanical low back pain, but there is no single study available in the literature on the combined effects of manual physical therapy and core stability exercise therapy. The objective of the current study was to determine the combined effect of manual physical therapy and core stability exercise therapy in the management of mechanical low back pain. ^7^

The manual therapy is a commonly used management of mechanical low back pain to improve the mobility of the lumbar spine. These techniques are based on glides passively procured in prone position by manual physical therapists. The techniques used in this research study were central anterior-posterior glides, and unilateral anterior-posterior glides. The aim of specific lumber mobilization techniques is to improve pain and increase mobility ^8^. 

The core stabilization exercises for strengthening of spinal muscles to improve their ability to maintain neutral spine using the abdominal, back, neck and shoulder girdle muscles as stabilisers rather than movers. There are two types of core stability exercises; the static activities exercises and dynamic floor exercise. Here in this research study we use Plank, Side plunk, Bridge, and Superman as static activities core stabilization exercise and Side lying with abduction, Oblique crunch, Straight leg raise, and Lying wind screen as dynamic floor core stability exercises ^9^. (Table-I)

## METHODS

A 6 month pretest-posttest design, quasi experimental study was conducted at department of physiotherapy Khyber Teaching Hospital (KTH) Peshawar, Pakistan. We conveniently selected a sample 40 patients and placed into two groups. The SLM techniques with CS exercises was applied in group A and CS exercises alone in group B for 6 weeks. The Oswestry Disability Index (ODI) and Visual analog scale (VAS) for mechanical low back pain were assessment tools assessed for all patients before and after 6 weeks of physical therapy intervention. Data was analyzed by SPSS and statistical test were applied at 95% level of significance determine the efficacy of both the treatments regimes and compared with each other. 

All the 40 patients were treated for 6 weeks at 4 days per week, for single session of 45 minutes. The specific lumber mobilization techniques ( central antero-posterior -CPA, and unilateral antero-posterior-UPA) were applied in prone lying position at 6-8 glides per session from T12 to L5 and followed by core stability exercises for local muscles (multifidus, transverse abdominis, diaphragm, pelvic floor muscles) and global dynamic muscles (rectus abdominis, internal oblique, external oblique, erector spine) muscles. The physical activities for these core stability exercises are listed in table-I and all the activity positions were maintained for 5-10 seconds for 10 repetitions. The specific joint mobilization techniques were applied in group B alone. 

## RESULTS

The base line characteristics of all 40 patients were documented before the start of the study (Table-II). All the 40 patients with low back pain underwent through a 6 week physical therapy management. The result of this study shows that the mean pain intensity was 6/10 and disability score 36 on ODI (moderate disability) before intervention in group A, which was treated by specific lumber mobilization and core-stabilization exercises improve pain intensity to 2/10 on VAS and disability score to 16 (minimal disability). 

The mean pain intensity was 7/10 and disability score 38 on ODI (moderate disability) before intervention in group B, which was treated by specific lumber mobilization techniques alone improve pain intensity to 5/10 on VAS and disability score to 26 (moderate disability).

After comparison between two groups, the group A treated with specific lumber mobilization techniques shows better results in improving pain (p=0.008) and reducing physical disability (p=0.004) as compared to the group B treated with specific lumber mobilization techniques alone (pain intensity: p= 0.172 and physical disability: p=0.201). (Table-III)

## DISSCUSSION

The result of this research study shows that the specific lumber mobilization techniques combined with core stabilization exercises are in the management of Mechanical low back pain can manage better manage pain and disability, as compared with lumber specific mobilization alone. Delitto and colleagues developed the clinical practice guidelines on low back pain under the orthopedic section of American Physical Therapy association. They recommended that the non-thrust mobilization improves pain, spinal mobility, and disability in both acute and chronic pain and pain related to lower extremity. These recommendations are based on strong evidence. ^10^

**Table-I:** static and dynamic activates for stability exercises

*Static Activities*	*Dynamic Floor Exercises*
Plank	Side lying with abduction
Side plunk	Oblique crunch
Bridge	Straight leg raise
Super man	Lying wind screen

**Table-II:** Baseline characteristics of all 40 patients of Mechanical low back pain (MLP)

*Characteristic*	*Group A*	*Group B*	*Total*
Male patients	15(75%)	17(85%)	32 (80%)
Female patients	05(25%)	03(15%)	8 (20%)
Sedentary life style	12(60%)	11(55%	23(57%)
Active life style	08(40%)	09(45%)	17(43%)
VAS 0-10 ( mean)	06.85	07.0	06.92
ODI 0-50 (mean)	23.4	26.0	24.70

**Table-III:** Clinical and functional changes in all 40 patients at six weeks in patients with Mechanical low back pain (MLP)

*Characteristic*	*Group A*			*Group B*		
	*Mean*	*Standard deviation*	*P-value*	*Mean*	*Standard deviation*	*P-value*
VAS	0.95	1.4+0.32	0.008	0.55	1.7+0.26	0.172
ODI	0.80	1.2+0.24	0.004	0.33	1.1+0.20	0.201

Salven and team conducted a systematic literature review on the relative effectiveness of segmental specific level and non-specific level spinal joint mobilization on pain and range of motion. They found multiple studies which provide evidence that single session of joint mobilization can lead to reduction in pain at rest and with most painful movements. ^11^

Aure and group conducted a randomized control trial to compare the effect of manual therapy with exercise therapy on 49 patients. They applied manual therapy on 27 and exercise therapy on 22 patients for the time course of 2 month. They concluded that the improvements in movement were more in manual therapy group as compare to the patient in the exercise therapy group. ^12^

Sarigiovannis and associates carried out a review to assess the evidence for the effectiveness of cervical spine manipulation and mobilization in the treatment of non-specific neck pain. The total of 12 randomized control trials meets the criteria and achieved the methodological score range from 25-67 out of 100. The available evidence assessed for these 12 randomized control trials supports the effectiveness of spinal manual therapy in non- specific neck pain while applied in conjunction with exercise therapy. ^13^

Xue-Qiang Wang and colleagues conducted a meta-analysis to review the effects of core stability exercise or general exercise for patients with chronic low back pain (LBP). They found 28 potentially relevant trials, which support the evidence for the effectiveness of core stability exercises as compare to the general exercises in patients with chronic low back pain. ^14^

Cairns and team carried out a pragmatic, multicentered randomized controlled trial on 97 patients of low back pain with 12 months follow up. All patients were placed into two groups through stratified random sampling technique. The conventional physical therapy including general active exercise combined with manual therapy was applied in group A, while conventional physical therapy combined with core stability exercise therapy in group B. sixty eight patients out of 97 completed the study and on the basis of results obtained they concluded that both packages of treatment showed the same effects and the sign and symptoms were improved with same degree in both treatment groups. ^15^

Valerie Gladwell and group conducted A single blind randomized controlled trial to evaluate the effect of a program of modiﬁed Pilates for active individuals with chronic non-speciﬁ c low back pain on 49 patients of low back pain. All the patients were randomly placed into two group and group A 24 patients was the Pilates group and active population group includes 25 patients. Total of 34 patients completed the study, 14 in group A and 20 in group B. they concluded that Pilates used as a speciﬁ c core stability exercise incorporating functional movements can improve non-speciﬁ chronic low back pain in an active population compared to no intervention.^16^

## CONCLUSION

It is concluded that patients with mechanical low back pain will show more improvement in pain and function while treated by specific lumber mobilization and core stability exercises as compared to those patients who will be treated by specific joint mobilization techniques.

## Authors contribution:

Dr. Rafiq Ahmed and Dr. Syed Shakil-ur-Rehman conceived, designed and did statistical analysis & editing of manuscriptDr. Rafiq Ahmed, Dr. Syed Shakil-ur-Rehman, and Dr Fozia Sibtain did data collection and manuscript writing
